# Visualization of real-time receptor endocytosis in dopamine neurons enabled by NTSR1-Venus knock-in mice

**DOI:** 10.3389/fncel.2022.1076599

**Published:** 2022-11-29

**Authors:** Aliza T. Ehrlich, Pierre Couvineau, Selin Schamiloglu, Stefan Wojcik, Dillon Da Fonte, Amina Mezni, Mark von Zastrow, Kevin J. Bender, Michel Bouvier, Brigitte L. Kieffer

**Affiliations:** ^1^Douglas Research Centre, Department of Psychiatry, McGill University, Montreal, QC, Canada; ^2^Department of Psychiatry and Behavioral Sciences, University of California, San Francisco, San Francisco, CA, United States; ^3^Institut de Recherche en Immunologie et en Cancerologie (IRIC), University of Montreal, Montreal, QC, Canada; ^4^Neuroscience Graduate Program, University of California, San Francisco, San Francisco, CA, United States; ^5^Department of Neurology, Kavli Institute for Fundamental Neuroscience, Weill Institute for Neurosciences, University of California, San Francisco, San Francisco, CA, United States; ^6^INSERM U1114, University of Strasbourg, Strasbourg, France

**Keywords:** G protein-coupled receptor (GPCR), brain, trafficking, neurotensin, PD149163

## Abstract

Dopamine (DA) neurons are primarily concentrated in substantia nigra (SN) and ventral tegmental area (VTA). A subset of these neurons expresses the neurotensin receptor NTSR1 and its putative ligand neurotensin (Nts). NTSR1, a G protein-coupled receptor (GPCR), which classically activates Gαq/calcium signaling, is a potential route for modulating DA activity. Drug development efforts have been hampered by the receptor’s complex pharmacology and a lack of understanding about its endogenous location and signaling responses. Therefore, we have generated NTSR1-Venus knock-in (KI) mice to study NTSR1 receptors in their physiological context. In primary hippocampal neurons, we show that these animals express functional receptors that respond to agonists by increasing intracellular calcium release and trafficking to endosomes. Moreover, systemic agonist administration attenuates locomotion in KIs as it does in control animals. Mapping receptor protein expression at regional and cellular levels, located NTSR1-Venus on the soma and dendrites of dopaminergic SN/VTA neurons. Direct monitoring of receptor endocytosis, as a proxy for activation, enabled profiling of NTSR1 agonists in neurons, as well as acute SN/VTA containing brain slices. Taken together, NTSR1-Venus animals express traceable receptors that will improve understanding of NTSR1 and DA activities and more broadly how GPCRs act *in vivo*.

## Introduction

G protein-coupled receptors (GPCRs) are the target of upwards of 30% of commercially available drugs (Hauser et al., 2017). Their membrane localization makes them accessible targets, but their low abundance can make them difficult to study in native systems. Developing tools that broaden our understanding of GPCR biology is integral to improving drug development efforts. Dopamine is a key neurotransmitter required for learning, reward and movement. Disrupted dopamine neurotransmission is involved in psychiatric diseases like schizophrenia and addiction. Dopamine D2 receptors are the primary pharmacological target of dopamine therapies. Chronic use of D2 targeted therapies can have adverse effects such extrapyramidal effects, abnormal involuntary movements, hallucinations, or altered mood (Beaulieu and Gainetdinov, 2011; Torti et al., 2019). Thus, developing alternate pharmacological methods to regulate dopamine is of high importance.

Neurotensin (NTS) is a neuropeptide that modulates many physiological processes including dopamine neurotransmission, body temperature, blood pressure, feeding, analgesia, and locomotion (Mustain et al., 2011; Torruella-Suarez and McElligott, 2020). NTSR1 is expressed in ventral tegmental area (VTA) and substantia nigra (SN) dopamine neurons (Uhl et al., 1977; Boudin et al., 1996; Schroeder and Leinninger, 2018), and many of its physiological actions are thought to be mediated through effects on dopamine neuromodulation. NTSR1 agonists have been shown to decrease drug-seeking behaviors positioning these receptors as potential targets for developing addiction treatments (Sharpe et al., 2017). In fact, recently described allosteric modulation and β-arrestin biased signaling of NTSR1 (Peddibhotla et al., 2013; Barak et al., 2016; Slosky et al., 2020) open new possible strategies to mitigate addiction. Additionally, NTSR1 holds unique pharmacology, evidenced by its pleiotropic signaling in recombinant systems (Besserer-Offroy et al., 2017) and heterodimer signaling with dopamine D2 and D3 receptors (Koschatzky et al., 2011; Borroto-Escuela et al., 2013), which can be specifically targeted by innovative bivalent ligands (Budzinski et al., 2021). Thus, neurotensin receptors are promising targets for regulating dopamine neurotransmission. However, how the subcellular organization of NTSR1 trafficking and activation contributes to dopamine neuromodulation is not well-understood.

Current animal models that have enabled NTSR1 study include NTSR1-KO mice. Loss of NTSR1 function established a role in dopaminergic reward, managing features associated with schizophrenia (Mechanic et al., 2009; Liang et al., 2010), and established a lack of involvement in analgesia which is mediated through NTSR2 (Pettibone et al., 2002; Remaury et al., 2002; Maeno et al., 2004; Mechanic et al., 2009). Additionally, the Gene Expression Nervous System Atlas (GENSAT) project created the NTSR1-EGFP transgenic animals which express EGFP in NTSR1 targeted neuronal populations (Gong et al., 2003) and *Ntsr1-Cre* animals which display Cre-recombinase expression in NTSR1 targeted cell populations (Gong et al., 2007). The GENSAT animal models enable the close examination of NTSR1 circuitry. However, high-resolution monitoring of activation and trafficking of NTSR1 itself cannot be investigated with currently available tools.

Here, we introduce a new animal model that allows for the study of NTSR1 receptor activity by directly monitoring the receptor. NTSR1-Venus mice were generated by knocking-in a Venus fluorescent protein following the C-terminus of NTSR1 at the endogenous gene locus, using techniques successful in our previous fluorescent receptor knock-in animals (Scherrer et al., 2006; Erbs et al., 2015; Ehrlich et al., 2018, 2019, 2021). We previously found in fixed cell preparations, that measuring endocytosis of an opioid receptor fused to Venus is an informative and reliable way to profile drug activities (Ehrlich et al., 2019). As monitoring GPCR trafficking in living tissues is a highly desired yet unmet goal in the field, we reasoned that the same proxy (monitoring receptor endocytosis) could be used to monitor NTSR1 receptor activation in living cells. In the present study, we demonstrate that Venus tagging of NTSR1 is sufficient to enable monitoring subcellular location and trafficking of receptors in intact brain tissue. Together, our findings support NTSR1-Venus animals as a key investigative system to study GPCR activity in living tissues and propel the development of therapeutics in neuropsychiatry.

## Materials and methods

### Reagents

The compounds Ionomycin calcium salt from Streptomyces conglobatus, Neurotensin, PD149163 tetrahydrochloride hydrate, SR48692 were purchased from Sigma Millipore, St Louis, MO, USA. Phosphate Buffered Saline was purchased from ThermoFisher, Waltham, MA, USA.

### Plasmids

The mouse coding sequence of mouse Gαq was subcloned into pcDNA3.1 using Gibson assembly [New England Biolabs (NEB), Ipswich, MA, USA] with the following primers integrating the restriction sites NHE1-Xho1: mGq-Fwd: 5′-atacgactcactata gggagacccaagctggctagcgtttaaacttaagcttggtaccgc−3′. mGq-Rvs: 5′ -tccaccacactggactagtggatccgagctgaccagattgtactccttcaggttcagctg− 3′. The mouse coding sequence of NTSR1 was PCR amplified from pCMV6-mNTSR1 (Origene, Rockville, MD, USA) and subcloned upstream from the IRES feature with a STOP codon before IRES into pIRES (Invitrogen, Waltham, MA, USA) and pIRES-Venus vector previously described (Hamdan et al., 2005) resulting in expression of either untagged NTSR1 or NTSR1-Venus. Additionally, pcDNA3.1-RlucII vector previously described (Namkung et al., 2016) using Gibson assembly with the following primers integrating the restriction site *Bam*HI for both pIRES and pcDNA3.1: pIRES-mNTSR1-fwd: 5′-tatctgcggcctagctagccaccaggatccgccaccatgcacctcaacagctccgtgcagc aggga−3′ for both pIRES constructions. pIRES-mNTSR1-rvs: 5′-gcccttgctcaccatggtggcgatggatccttagtacagggtttcccgggtggcgctgg t−3′. pIRES-mNTSR1-Venus-rvs: 5′-gcccttgctcaccatggtggcgatg gatccgtacagggtttcccgggtggcgctggtgga−3′. pcDNA3.1-mNTSR1-RlucII-fwd: 5′-atagggagacccaagctggctagcggatccgccgccgcgatcgcc atggaagatgatggt−3′. pcDNA3.1-mNTSR1-RlucII-rvs: 5′ gat ggcgcgcccaccggtaccggcggatccgtccacaagggtttcttgactatagggaat−3′. The pIRES vector was used The rGFP-CAAX BRET and G_q_ GEMTA biosensors construct were generated as previously described (Namkung et al., 2016; Avet et al., 2022).

### Cell culture and transfections

HEK293 clonal cell line (HEK293SL cells), hereafter referred as HEK293 cells, were a gift from S. Laporte (McGill University, Montreal, Quebec, Canada) and previously described (Namkung et al., 2016). Cells were maintained in Dulbecco’s Modified Eagle Medium (DMEM, Wisent, Saint-Jean-Baptiste, QC, Canada) supplemented with 10% fetal bovine serum (FBS, Wisent) and 1% antibiotics [100 U/mL penicillin and 100 μg/mL streptomycin (PS); Wisent]. Cells were grown at 37°C in 5% CO2 and 90% humidity and checked for mycoplasma contamination. Two days prior to experiments, cells were trypsinized (Wisent) and 35,000 cells were transfected with 1 μg of total DNA containing the appropriate expression vectors/biosensors. The total quantity of DNA was completed at 1 μg with salmon sperm DNA (Invitrogen, Waltham, MA, USA). Transfection was performed using the transfecting agent polyethylenimine 25 kD linear (PEI; Polysciences, Warrington, PA, USA) at a ratio of 3:1 PEI/DNA. Cells were then immediately plated onto poly-ornithine (Sigma-Aldrich) coated 96-well white culture plates (PerkinElmer, Waltham, MA, USA).

### Bioluminescence resonance energy transfer measurement

Forty-eight hours post-transfection, cells were washed once with phosphate-buffered saline (PBS) then incubated 1 h at 37°C in Tyrode buffer (137 mM NaCI, 0.9 mM KCI, 1 mM MgCI2, 1 1.9 mM NaHCO3, 3.6 mM NaH2PO4, 25 mM HEPES, 5.5 mM Glucose, and 1 mM CaCI2, pH 7.4). Cells were then treated with the ligands and prior to measurement treated with the substrate (5 min with 2.5 μM Coelantarazine 400a). Bioluminescence resonance energy transfer (BRET) was then measured between RlucII (BRET energy donor) and rGFP (enhanced bystander ebBRET acceptor)–tagged proteins. BRET values were read for 1 s per well using a Mithras™ LB940 Multimode Microplate Reader (Berthold Technologies, Bad Wildbad, Germany) for concentration–response curves. ebBRET values were obtained by calculating the ratio of the light emitted by the energy acceptor over the light emitted by the energy donor (donor 410 ± 70 nm/acceptor 515 ± 20 nm). Data were collected using the MicroWin 2000 software (Berthold Technologies, Bad Wildbad, Germany). They were then fitted and analyzed in GraphPad Prism (v9.0, GraphPad Software, Inc., San Diego, CA, USA).

### Animals

The NTSR1-Venus mouse line was established by PHENOMIN at the MCI/ICS [Mouse Clinical Institute–Institut Clinique de la Souris (ICS), Illkirch, France^1^ ]. The targeting vector was constructed as follows (Figure 2A). A 3.5 kb fragment encompassing exon 2, intron 2, exon 3, and partial intron 3 of *Ntsr1* was amplified by PCR (from RP23-299O18 BAC containing *Ntsr1*) and subcloned in an ICS proprietary vector. This ICS vector bares a floxed Neomycin resistance cassette associated with a Cre autoexcision transgene that will allow the excision of the whole cassette in the chimera’s male germ line. Three fragments of 784 bps for the linker-Venus fragment, 730 bps for the partial intron 3-exon 4 fragment and 3619 bps for the end of exon 4 (3′UTR) and surrounding genomic region were amplified by PCR and subcloned by SLIC cloning in step 1 plasmid to generate the final targeting construct. The linearized construct was electroporated in C57BL/6N mouse embryonic stem (ES) cells (ICS proprietary line). After G418 selection, targeted clones were identified by long-range PCR and further confirmed by Southern blot with an internal (Neo) probe and a 5′ external probe. Two positive ES clones were validated by karyotype spreading and microinjected into BALB/C blastocysts. Resulting male chimeras were bred with wild type C57BL/6N females. Germline transmission with the direct excision of the selection cassette was achieved in the first litter.

**FIGURE 1:**
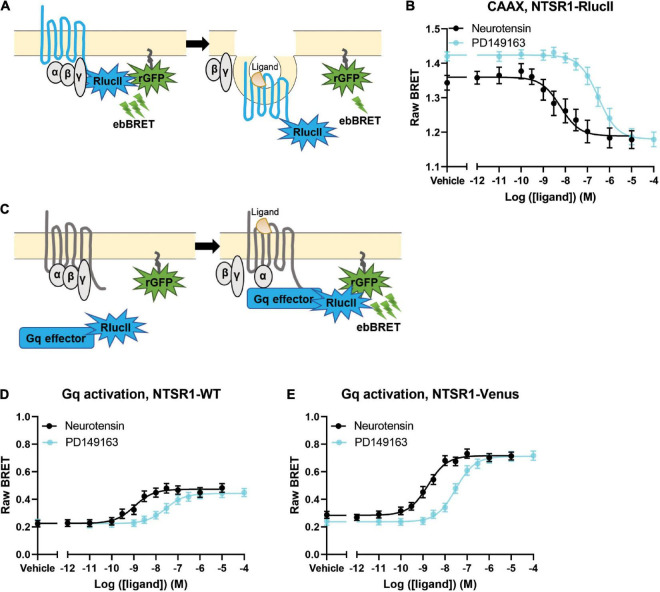
Agonist profiling of NTSR1 in HEK-293 cells. **(A)** Scheme for BRET assay to measure receptor loss from the membrane shows receptor tagged with RlucII as the donor and rGFP-CAAX labeling the membrane as the acceptor. A decrease in BRET indicates receptor loss from the plasma membrane. **(B)** Dose-response curves for NTSR1-RlucII internalization, using NTSR1-RlucII (donor) and rGFP-CAAX (acceptor), profile two agonists, neurotensin and PD149163. **(C)** Scheme for BRET assay to measure agonist induced G protein activation shows Gq effector (Gq/11 binding domain of p63-RhoGEF fused to RlucII) tagged with RlucII as the donor and rGFP-CAAX labeling the membrane as the acceptor. An increase in BRET indicates G protein activation. **(D,E)** Gq protein activation BRET assay compares dose-response curves for neurotensin and PD149163 at the untagged NTSR1 **(D)** and at the Venus tagged **(E)** NTSR1. Data are expressed as raw BRET signal (Mean ± SEM) from five independent experiments.

**FIGURE 2:**
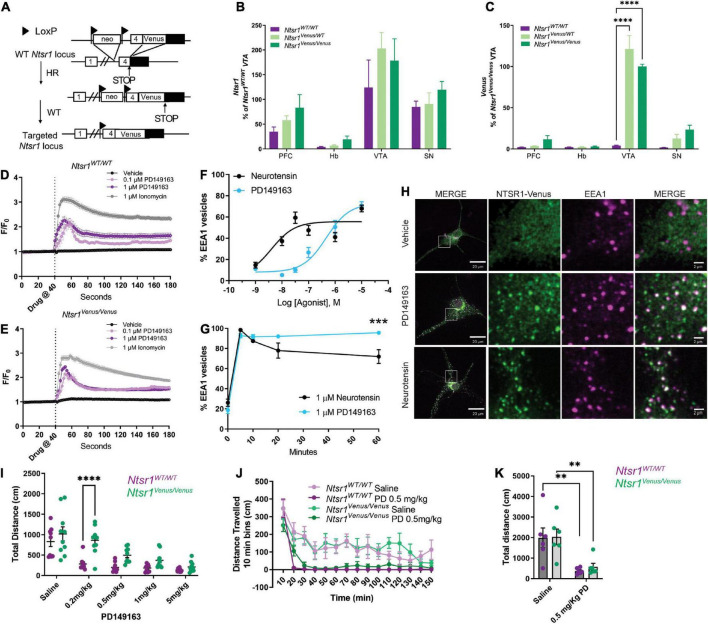
NTSR1-Venus knock-in mouse generation and functional characterization. **(A)** Scheme of NTSR1-Venus knock-in mouse generation. Briefly, *Venus* was knocked-in at the C-terminus of *Ntsr1* at the endogenous gene locus. **(B,C)** mRNA transcript expression is shown across mouse brain regions–prefrontal cortex (PFC), habenula (Hb), ventral tegmental area (VTA), and substantia nigra (SN) in *Ntsr1^WT/WT^*, *Ntsr1*^KI/WT^**, and *Ntsr1^KI/KI^* (*n* = 3–5 animals per group). **(B)** *Ntsr1* gene expression. **(C)** *Venus* gene expression. ^****^p < 0.0001 *via* 2way ANOVA with a Dunnett’s *post-hoc* test [significant effect by brain region *F*_(3_,_39)_ = 78.35; *p* < 0.0001; significant effect by genotype *F*_(2_,_39)_ = 30.01; *p* < 0.0001, significant interaction *F*_(6_,_39)_ = 18.65; *p* < 0.0001]. **(D,E)** Intracellular Ca2+ response was measured in hippocampal neurons from either *Ntsr1^WT/WT^* **(D)** or *Ntsr1^Venus/Venus^* **(E)** mice. Hippocampal neurons were treated with vehicle, 0.1 μM, 1 μM PD149163, or 1 μM Ionomycin. Drug was added at 40 s. *n* = 11–148 cells per condition, each cell was normalized to baseline for the individual cell. **(F–H)** Agonist profiling by monitoring internalization of NTSR1-Venus in hippocampal primary neurons from *Ntsr1^Venus/Venus^* mice treated with 1 μM neurotensin or PD149163. The % EEA1 vesicles was calculated by taking the ratio of Venus to EEA1 puncta. **(F)** Dose-response curves. Neurons were treated with the indicated concentrations of PD149163 or neurotensin and fixed after 5 min. *n* = 20–23 neurons per condition. **(G)** Internalization time-course. Hippocampal neurons from *Ntsr1^Venus/Venus^* mice were treated with 1 μM PD149163 or neurotensin and fixed at indicated timepoints. 0 min received no treatment. *n* = 13 neurons per condition. ^***^p = 0.0002 *via* 2way ANOVA with a Sidak’s *post-hoc* test [significant effect by time *F*_(4_,_111)_ = 122.1; *p* < 0.0001], significant effect by drug *F*_(1_,_111)_ = 5.350; *p* = 0.0226 with a significant interaction *F*_(4_,_111)_ = 5.690; *p* = 0.0003. **(H)** Representative images of NTSR1-Venus internalization in hippocampal neurons from *Ntsr1^Venus/Venus^* mice. Cells were treated with vehicle, or NTSR1 agonists 1 μM PD149163 or neurotensin for 60 min and fixed and stained with anti-Venus (green) or EEA1 (magenta). White boxes indicate 10x magnified area. Scale bars are 20 μm for the macro view and 2 μm for the magnified view. **(I)** 30 minutes following *i.p.* injection of NTSR1 agonist, PD149163, or saline dose-response locomotor activity effects were assessed by measuring horizontal movement (cm) during 150 min in activity boxes. *n* = 9 or 10 for *Ntsr1^WT/WT^* and *Ntsr1^Venus/Venus^* mice, respectively. ^****^p < 0.0001 for 0.2 mg/kg *Ntsr1^WT/WT^* vs. *Ntsr1^Venus/Venus^ via* 2way RM ANOVA Sidak’s multiple comparisons test [Dose significant effect *F*_(4_,_68)_ = 31.61; *p* < 0.0001, Genotype significant effect *F*_(1_,_17)_ = 12.91; *p* = 0.0022, significant interaction *F*_(4_,_68)_ = 3.242; *p* = 0.0170]. **(J)** Locomotor activity was measured 30 min following *i.p.* injection of saline or 0.5 mg/kg PD149163 in tagged and untagged NTSR1 animals. Data shown in 10 min bins of distance traveled. 2way ANOVA Sidak’s multiple comparisons test was not significant for genotype effect [*F*_(1_,_10)_ = 0.005494; *p* = 0.9424 saline, *F*_(1_,_10)_ = 1.261; *p* = 0.2877 PD 0.5 mg/kg]. **(K)** Total distance traveled in animals treated with 0.5 mg/kg PD149163 compared to saline treated animals. *n* = 6 for *Ntsr1^Venus/Venus^* and *Ntsr1^WT/WT^* each. ^**^p < 0.01 *via* 2way ANOVA Sidak’s multiple comparisons test [Saline vs. 0.5 mg/kg, *p* = 0.0038 and *p* = 0.0087 for *Ntsr1^WT/WT^* and *Ntsr1^Venus/Venus^* respectively, Drug significant effect *F*_(1_,_20)_ = 23.01; *p* = 0.0001, Genotype effect *F*_(1_,_20)_ = 0.1537; *p* = 0.6992]. Graphs depict the mean ± SEM.

*Ntsr1^Venus/WT^* mice were intercrossed to generate *Ntsr1^Venus/Venus^* mice, which are fertile and develop normally. *Ntsr1^WT/WT^*, *Ntsr1*^Venus/WT^**, and *Ntsr1^Venus/Venus^* mice were bred in-house (Neurophenotyping Center, McGill University/Douglas Hospital Research Institute, Montreal, Canada) on a C57Bl/6N (Charles River Laboratories, Wilmington, MA, USA) background. Animals were group-housed and maintained on a 12-h light/dark cycle (lights on at 8:00 a.m.) at a controlled temperature (22 ± 1°C) and humidity (45 ± 5%). Food and water were available *ad libitum* throughout all experiments, unless otherwise stated. Male and female mice between 8 and 14 weeks were used for immunohistochemistry, and male mice 8–16 weeks were used for behavior experiments. All experimental procedures were performed in accordance with the guidelines of the Canadian Council of Animal Care, and all animal procedures were approved by the McGill University/Douglas Hospital Animal Care Committee. All animals were routinely genotyped at weaning and after experimental endpoints using the following genotyping primers: Forward 5′-GCTAAGCATGACTCAGGCTCCCAG-3′ Reverse 5′-GATCTTAACTCCCATCTCCAGGCAAC-3′ which produced the following sized bands for each genotype: bp 299–*Ntsr1^WT/WT^*, 394 bp—*Ntsr1^Venus/Venus^* mice, both bands are visible for *Ntsr1^Venus/WT^*. For multiphoton experiments, animals were bred and housed at UCSF and cared for by Laboratory Animal Resource Center Staff following Institutional Animal Care and Use Committee approved protocol (AN185688).

### RNA sample preparation and isolation

RNA was collected using freshly dissected brains on ice, brains were severed along the midline and hemispheres were immediately frozen on dry ice and stored at −80°C until use. TRIzol (Invitrogen, Waltham, MA, USA) reagent was added directly into the collected sample tubes. Mechanical dissociation was applied to yield a homogenate and tissue extraction was done according to the manufacturer’s protocol. The precipitate carrier, Dr. Gentle (Takara, Kusatsu, Shiga, Japan), was added to visualize RNA pellets. RNase-and DNase- free water was used to resuspend the Total RNA (ThermoFisher, Waltham, MA, USA). Purity and concentration of total RNA was evaluated on a NanoDrop ND2000 (ThermoFisher, Waltham, MA, USA).

### RT-qPCR

RT-qPCR was performed as described previously (Ehrlich et al., 2019, 2021). M-MLV Reverse Transcriptase Kit (Invitrogen, Waltham, MA, USA) reverse transcribed 400 ng of RNA according to the manufacturer’s instructions. cDNA was diluted 2 times in RNase- and DNase-free water. 2 μl of cDNA together with forward and reverse primers (0.5 μM each primer) and 5 μl of LightCycler 480 SYBR I Green Master Mix (Roche, Basel, Switzerland) was used. cDNA samples were loaded in 384-well white polypropylene plates (Roche, Basel, Switzerland) in triplicates. Samples were run for 45 cycles of amplification on the LightCycler 480 II Real-Time PCR System (Roche, Basel, Switzerland). Water, in place of cDNA, served as a no template control reaction to check for non-specific amplification. *Tyrosine hydroxylase* (Th) expression was used to validate VTA specific samples. Samples with low *Th* expression were excluded from the analysis. The average of housekeeping gene *B2m*, was subtracted from the average of the triplicate CT values for each sample. Relative fold changes were obtained using the comparative CT method (2^–ΔΔCT^) (Livak and Schmittgen, 2001) and multiplied by 100 to show the data in percent.

### Table of RT-qPCR primers

**Table:** 

Gene	Forward	Reverse
*Venus*	CACATGAAGCAGCA CGACTT	CATTGTGGGCGTTGT AGTTG
*Ntsr1*	GCAGCCGCACCAAG AAATTCA	ATGAAGGTGTTAACCT GGATGACGA
*B2m*	TGGTGCTTGTCTCACT GACC	GTATGTTCGGCTTCCC ATTC
*Th*	CCTGGAGTACTTTGTG CGCT	GGGAACCAGGGAACCT TGTC

### Preparation of NTSR1-Venus hippocampal neuronal cultures

To isolate hippocampal neurons, P0 pups were removed from the cage of NTSR1-Venus homozygous knock-in breeding pairs. Pups were decapitated, brains were removed dorsal side up into fresh ice-cold Hank’s balanced salt solution (HBSS; Gibco, Grand Island, NY, USA), meninges were removed, and hippocampi were dissected into Hibernate A (Gibco, Grand Island, NY, USA) on ice. Tissue dissociation was carried out with the Papain Dissociation System according to the manufacturer’s protocol (Worthington Biochemical Corporation, Lakewood, NJ, USA). Neurons were plated on Poly-D-Lysine (Sigma) coated surfaces at 100,000 cells per 12 mm coverslip (NeuVitro, Camas, WA, USA) in a 24-well plate for immunostaining or 50,000 cells per well of an 8 well chamber slide (Ibidi, Grafelfing, Germany) for calcium measurements. Plating media [Neurobasal A (Gibco, Grand Island, NY, USA), 1% FBS (Fisher Scientific), 2% B-27 (Gibco, Grand Island, NY, USA), 0.5 mM GlutaMAX (Gibco, Grand Island, NY, USA), and 1x Penicillin/Streptomycin (Invitrogen, Waltham, MA, USA)] was exchanged 1 h after plating to maintenance media (Neurobasal A, 2% B-27, 0.5 mM GlutaMAX, and 1x Penicillin/Streptomycin). Half of the volume of media was exchanged for fresh media every 3–4 days. On day *in vitro* (DIV) 3 Cytosine arabinoside hydrochloride (Sigma) was added to media with a final concentration of 0.1 μM to decrease glial cell proliferation.

### Measurement of intracellular calcium release

Hippocampal neurons were grown until DIV 7. Fluo-4 NW Calcium Assay kit (Thermo Fisher) was used according to the manufacturer’s protocol. Briefly, drugs were prepared at 4 μM stock solutions in the assay buffer provided without probenecid. Growth media was removed and 150 μL of the dye loading solution was added to each well. Imaging dishes were incubated at 37°C without added CO_2_ for 30 min and then a further 30 min at room temperature. Fluorescence was imaged with a 10x objective on an (Olympus, Shinjuku City, Tokyo, Japan) IX73 microscope (Fluo-4 em. 506 nm).

Live imaging settings of Fluo-4 were as follows, 79 ms exposure, 2 s intervals for 90 cycles (180 s). 50 μL of drug for a final concentration of 1 μM was added at cycle 20 (40 s). Data analysis was performed using FIJI Image J as follows. First regions of interest (ROI) were automatically determined using the analyze particles plug in. Then integrated density was obtained for all ROIs across all 90 images. For each agonist treated well ∼200 cells were examined to identify cells with activation between 40 and 60 s. For vehicle treated wells, all Fluo4-loaded cells were included in the analysis. Relative fluorescence is defined as F/F_0_ where F corresponds to the cell fluorescence and F_0_ to the basal fluorescence.

### Monitoring NTSR1-Venus endocytosis in primary neurons

Hippocampal neurons from *Ntsr1^Venus/Venus^* animals were cultured on PDL coated coverslips (NeuVitro, Camas, WA, USA) in a 24 well plate until DIV7. Drugs were prepared in Neurobasal A without serum or antibiotics. 1 mL of drug or vehicle containing media was added to the neurons and they were returned to the incubator for the indicated times (0, 5, 10, 20, or 60 min). All media was aspirated, and neurons were fixed in 4% paraformaldehyde (PFA; Electron Microscopy Sciences, Hatfield, PA, USA)/PBS pH 7.4 (ThermoFisher, Waltham, MA, USA) for 10 min at room temperature. Permeabilization followed in 0.1% Triton X-100/PBS (Sigma-Aldrich) for 10 min at room temperature. Coverslips were blocked in 1x PBS, 3% NGS, and 0.2% Triton X-100 (Sigma-Aldrich) for 1 h at room temperature. Primary antibodies chicken Anti-Venus (GFP, Novus, St Louis, MO, USA, 1:2000) and rabbit Anti-EEA1 (Cell Signaling Technology, Danvers, MA, USA, 1:1000) were diluted with blocking buffer and incubated overnight. Three washes with 0.1% Triton X-100/PBS were followed by secondary antibody incubation for 1 h at room temperature (Alexa 488 Anti Chicken 1:2000—Life Technologies and Alexa 647 Anti-Rabbit 1:2000—Life Technologies). Coverslips were washed in PBS and then incubated in DAPI (ThermoFisher, Waltham, MA, USA)/PBS and washed in PBS before mounting with Prolong Gold (ThermoFisher, Waltham, MA, USA) and drying overnight. An Olympus FV1200 confocal microscope with oil-immersion 60 × objective, was used to take z-stack images with laser settings–Alexa Fluor 488 Ex. 488/Em. 520 Alexa Fluor 647 Ex. 650/Em. 668.

For live imaging experiments, DIV6 neurons cultured on 8 well slide chambers (ibidi, Grafelfing, Germany) were transferred to incubators at the ABIF Imaging facility McGill University to acclimate overnight. DIV7 neurons were imaged at room temperature in phenol-red free cell imaging media (Thermo Fisher) with a 63x objective (Zeiss, Oberkochen, Germany) on an LSM710 microscope (Leica, Wetzlar, Germany). Venus was detected at Ex. 514 nm/Em. 528 nm. Microscope was set as follows, Line average 4, Gain 900, Zoom 2 with 3.87 s scan time. Movies were taken for 20 min with 30 s intervals. Drug or vehicle was diluted in cell imaging media and was added at 1 min.

### Immunohistochemistry on *Ntsr1-Venus* brain sections

Three animals of each genotype, homozygous wild type *Ntsr1-^WT/WT^*, heterozygous knock-in *Ntsr1-^Venus/WT^*, and homozygous knock-in *Ntsr1-^Venus/Venus^* were perfused at around 8 weeks old. The mice were anesthetized with 100 μl/100 g i.p. rodent cocktail mixture (ketamine, xylazine, acepromazine) intracardially cleared with ice cold 1x PBS, followed by 5 min with ice-cold 4% PFA (Cedarlane, Burlington, ON, Canada) using a peristaltic pump at 10 ml/min. The brains were then extracted and post-fixed in 4% PFA for 24 h, at 4°C. Then, the samples were cryo- protected in 30% sucrose (Thermo Fisher) for 24–48 h. Finally, the brains were stored in OCT (Thermo Fisher) at −80°C until processing. Brains were sliced on a Leica CM3050s cryostat at 30 μm thick, coronally, and stored free floating in 1x PBS at 4°C for storage. Sections were then washed in PBS-T (0.1% Triton X-100) to permeabilize the tissue, before blocking with 3% normal goat serum for 1 h at room temperature. Primary antibodies used included Chicken Anti-Venus (GFP, Novus, St Louis, MO, USA, NB100-16-17, 1:2000), Rabbit Anti-Tyrosine Hydroxylase (Abcam, Cambridge, UK AB112, 1:1000), Rabbit Anti-Dopamine-Beta-hydroxylase (Immunostar, Hudson, WI, USA 22806, 1:400), and were applied and incubated overnight at 4°C. There were 3 washes in PBS-T before addition of secondary antibodies; Goat Anti-Chicken Alexafluor 488 (Life Technologies A11039, 1:2000), Goat Anti- Chicken Alexafluor 594 (Life Technologies A11042, 1:2000), and Goat Anti-Rabbit Alexafluor 594 (Life Technologies A11012, 1:2000). These were incubated for 2 h at room temperature. The secondary antibody was then removed, before washing was done with PBS-T, followed by a PBS + DAPI wash to stain nuclei before a final wash in PBS and storage at 4°C before mounting.

For subcellular localization of NTSR1-Venus, confocal images were acquired on a laser scanning Olympus FV1200 microscope (Olympus Corporation, Shinjuku City, Tokyo, Japan) at 20x and 40x for higher magnification images. All images were acquired as Z-stacks and maximum projections of the original Z-stack are shown. Confocal imaging was done with the following laser settings, Alexa Fluor 488 Ex. 488/Em. 520, Venus Ex. 518/Em. 543, Alexa Fluor 594 Ex. 543/Em. 618.

For whole brain imaging, fixed, permeabilized, and stained 30 μm coronal sections were taken at intervals of 360 μm. Sections were imaged on an Olympus VS120 slide scanner at 10x objective Intrinsic Venus detected using FITC (Ex. 490 nm, Em. 520 nm) and amplified Venus detected using TRITC (Ex. 535 nm, Em. 590 nm). To map the expression of NTSR1-Venus, 92 brain regions were assessed for NTSR1-Venus according to the Allen Brain Interactive Atlas Viewer^2^ categorization. NTSR1-Venus expression level was assessed using the amplified signal (Anti-Venus). Exposure was first adjusted based on the region of highest signal (VTA) and the same scaled exposure setting was applied to each section. Qualitative expression analysis was ranked in 4 categories, none (0), low (1), moderate (2), and high (3). The final regional expression score is representative of the average of the expression observed in three independent *Ntsr1^Venus/Venus^* animals.

3D reconstructions of whole brain were done as follows. Images taken on a slide scanner (Olympus, Shinjuku City, Tokyo, Japan) were imported to FIJI (Schindelin et al., 2012) #3981 using the Olympus viewer plugin (Olympus, Shinjuku City, Tokyo, Japan) and concatenated into a stack. The individual sections were then imported into photoshop (Adobe, San Jose, CA, USA) to align them in 3D format and remove any artifacts manually and then exported back into FIJI for 3D reconstruction. The stack was then converted into a 3D stack with the 3D projection plugin with a 50 pixel gap on a 1024 × 1024 canvas and exported as .avi.

### Multiphoton acute slice experiments

Male and female *Ntsr1^Venus/Venus^* mice aged 5 weeks were anesthetized, and 250 μm-thick coronal slices containing substantia nigra pars compacta and ventral tegmental area were prepared. Cutting solution contained (in mM): 87 NaCl, 25 NaHCO_3_, 25 glucose, 75 sucrose, 2.5 KCl, 1.25 NaH_2_PO_4_, 0.5 CaCl_2_, and 7 MgCl_2_; bubbled with 5% CO_2_/95%O_2_; 4°C. Following cutting, slices were incubated in the same solution for 30 min at 33°C. For control experiments, slices were subsequently incubated in the same solution at room temperature until imaging. For antagonist experiments, slices were subsequently incubated in cutting solution with 1 μM SR48962 until imaging. SR48962 was prepared as a 1 mM stock in water.

Imaging solution referred to as aCSF contained (in mM): 125 NaCl, 2.5 KCl, 2 CaCl_2_, 1 MgCl_2_, 25 NaHCO_3_, 1.25 NaH_2_PO_4_, 25 glucose; bubbled with 5% CO_2_/95% O_2_; 31–34°C, ∼310 mOsm. For antagonist-only experiments, 1 μM SR48962 was added to the imaging solution. For antagonist plus agonist experiments, a baseline image was taken with no NTSR1 agonist or antagonist in the imaging solution, and subsequently, 10 μM PD149163 [prepared from a stock solution in water] was added to the imaging solution. As a precautionary measure, all components that were exposed to antagonist were rinsed first with 70% ethanol and then water. All components that were exposed to agonist were replaced between experiments.

For two-photon laser scanning microscopy (2PLSM), a 2-photon source (Coherent Ultra II, Santa Clara, CA, USA) was tuned to 920 nm for imaging. Epi- and transfluorescence signals were captured through a 60x, 1.0 NA objective, paired with a 1.4 NA oil immersion condenser (Olympus, Shinjuku City, Tokyo, Japan). Epifluorescence was acquired without bandpass filtering; transfluorescence was captured through a 550/49 nm bandpass filter (Semrock). Both epi and transfluorescence were detected with R9110 photomultiplier tubes (Hamamatsu, Shizuoka, Japan). Laser power measured at the focal plane under the objective was between 8 and 34 mW. NTSR1 localization images were acquired through Z-stacks taken in 1 μm steps every 15 min.

### Behavioral experiments

Locomotor activity was measured using Plexiglas activity boxes (20 × 20 × 20 cm) surrounded by horizontal and vertical infrared sensor beams (Versamax Omnitech Electronics, Columbus, OH, USA) during the light phase. The total numbers of animals used in locomotion experiments were *n* = 16 *Ntsr1^Venus/Venus^* and *n* = 15 *Ntsr1^WT/WT^* mice. Animals were habituated to the room in singly housed cages with home bedding for 1 h, before being injected with either 0.9% saline, 0.2, 0.5, 1, 5 mg/kg, PD149163, dissolved in 0.9% saline, and returned to their home cages for 30 min. Then mice were placed in horizontally opposed chambers, and horizontal movement (cm) was measured over the course of 2 h, in bins of 10 min.

### Statistical and image analysis

All images were viewed and analyzed using FIJI ImageJ (Schindelin et al., 2012). Statistical comparisons were carried out using GraphPad Prism. For BRET assays, Mann–Whitney unpaired T tests were used. For qPCR 2way ANOVA with Dunnett’s multiple comparisons test was used. Endocytosis in neurons and locomotor assays were analyzed by 2way ANOVA with Sidak’s or Bonferroni’s multiple comparisons test. Endocytosis in acute slices was analyzed by 2way ANOVA with Tukey’s multiple comparisons test. Differences were considered significant if *P* was less than 0.05.

## Results

### Generation of enhanced bystander bioluminescence resonance energy transfer constructs to assay NTSR1 activities

We first compared the ability of both neurotensin and PD149163 to induce NTSR1 internalization. We set-up an enhanced bystander bioluminescence energy resonance transfer (ebBRET) assay by fusing RlucII at the NTSR1 C-tail and expressing this construct together with rGFP-CAAX in HEK293 cells (Figure 1A). Using this assay, we monitored the NTSR1-RlucII internalization induced by both neurotensin and PD149163 by measuring the decrease in BRET between the RlucII donor and the rGFP acceptor (Figure 1B). Both compounds were able to induce NTSR1 internalization with significantly different potency (5.89 nM and 307 nM for neurotensin and PD149163, respectively, *p* = 0.0079) and efficacy (−0.180 and −0.245 for neurotensin and PD149163, respectively, *p* = 0.0159; Table 1).

**TABLE 1:** Efficacies and potencies of NTSR1 agonists on receptor internalization and Gq activation.

	NTSR1-RlucII internalization			

	Potency [LogEC50 (M)]		Efficacy (Raw BRET)	
2-3 Neurotensin	−8.070 ± 0.184		−0.180 ± 0.010	
PD149163	−6.513 ± 0.059		−0.245 ± 0.008	

	**Gq activation**			

	**NTSR1**		**NTSR1-Venus**	
		
	**Potency [LogEC50 (M)]**	**Efficacy (Raw BRET)**	**Potency [LogEC50 (M)]**	**Efficacy (Raw BRET)**

Neurotensin	−8.968 ± 0.041	0.248 ± 0.006	−8.783 ± 0.055	0.435 ± 0.009
PD149163	−7.552 ± 0.048	0.220 ± 0.013	−7.515 ± 0.034	0.476 ± 0.009

Potencies (LogEC50 in M) and efficacies (Raw BRET) of NTSR1 internalization and Gq activation represent mean ± SEM from five independent experiments performed in triplicate.

To test the functionality of the NTSR1-Venus construct used to generate the knock-in mice, the NTSR1 receptor was genetically fused to the fluorescent protein, Venus-YFP (Venus), at its C-tail. With another ebBRET assay, we compared its property to activate Gq with that of the wild-type (WT) NTSR1 by expressing in HEK293 cells either receptor, together with Gq effector membrane translocation assay; EMTA (Avet et al., 2022) effector, p63RhoGEF, tagged with RlucII and rGFP-CAAX. Using this assay, we monitored the increase in BRET transduction to assess the ability of both neurotensin and PD149163 to activate Gq, which leads to a relocalization of the Gq EMTA effector to the plasma membrane decorated on its intracellular face with rGFP-CAAX (Figure 1C). Neurotensin and PD149163 induced activation of Gq by both receptors (Figures 1D,E). For the untagged and WT receptors neurotensin and PD149163 have significantly different potencies to activate Gq with EC50s of 1.65 nM and 30.6 nM (*p* = 0.0079) respectively, for WT-NTSR1 and 1.08 nM and 28.0 nM (*p* = 0.0079), for NTSR1-Venus (Table 1). When we compared efficacy for neurotensin and PD149163 at each receptor, both compounds produced similar efficacies at a given receptor (0.248 and 0.220, respectively, for NTSR1 with *p* = 0.0556 vs. 0.435 and 0.476, respectively, for NTSR1-Venus with *p* = 0.222). However, the efficacies of the compounds at NTSR1-Venus were higher when compared to WT-NTSR1. Together, these data show that agonist-promoted receptor internalization and Gq activation are maintained for the C-tail modified NTSR1 (NTSR1-Rluc or NTSR1-Venus) with the expected potency differences between the compounds tested.

### Generation and functional characterization of NTSR1-Venus knock-in mice

Next, we created NTSR1-Venus knock-in mice to study NTSR1 localization and activity in native neurons and the brain. *Ntsr1^Venus/Venus^* animals were generated using homologous recombination (Figure 2A). A sequence encoding NTSR1 encompassing the linker-Venus fragment in frame with the open reading frame of NTSR1 at exon 4 was used to generate the targeting vector that included a floxed Neomycin resistance gene, which was later excised in the chimera’s male germ line (see Section “Materials and methods” for more details). To characterize the new animals, *Ntsr1* mRNA levels were measured in brain regions which were previously reported (Alexander and Leeman, 1998) to highly express *Ntsr1* and no significant alteration in *Ntsr1* levels was found between *Ntsr1^Venus/WT^* or *Ntsr1^Venus/Venus^* as compared to *Ntsr1^WT/WT^* animals (Figure 2B). *Venus* transcript levels were detected in a gene-dose-dependent fashion in the prefrontal cortex, ventral tegmental and substantia nigra areas but not detected in the habenula (Figure 2C). Although habenula had been previously determined to highly express *Ntsr1* using *in situ* hybridization in rat (Nicot et al., 1994; Alexander and Leeman, 1998), we failed to detect a high transcript level in this region for all genotypes of mice examined.

To examine NTSR1-Venus functionality, we evaluated NTSR1-Venus expression and agonist mediated redistribution in different neuronal preparations (hippocampal, cortical and midbrain; *data not shown*) and while NTSR1-Venus in all the preparations responded to the drug, we determined that expression was best detectable in hippocampal cultures. In mouse hippocampal neurons live imaged on an epifluorescent microscope, we first examined G protein signaling by measuring agonist-induced intracellular calcium levels using the calcium indicator, Fluo-4-AM. In both *Ntsr1^WT/WT^* (Figure 2D) and *Ntsr1^Venus/Venus^* (Figure 2E) neurons, vehicle did not elicit a calcium response while 1 μM Ionomycin, included to determine maximal calcium response in our experimental setting, induced a 3-fold calcium increase. 0.1 and 1 μM PD149163 induced a 2-fold intracellular calcium peak increase that was observed for *Ntsr1^WT/WT^* (Figure 2D) and *Ntsr1^Venus/Venus^* (Figure 2E) neurons indicating that the Venus fusion receptor can couple to Gq and elicit a calcium response.

Receptor endocytosis has often been used as a metric to distinguish drug activities. We next measured agonist-induced endocytosis in primary hippocampal cultures from *Ntsr1^Venus/Venus^* animals. Live confocal imaging of NTSR1-Venus neurons treated with 1 μM PD149163 showed robust internalization of the receptor (Supplementary Video 1). Whereas NTSR1-Venus remained diffuse across the plasma membrane in vehicle treated neurons (Supplementary Video 2). To quantify receptor endocytosis, hippocampal neurons were treated with either PD149163 or neurotensin for 5, 10, 20, or 60 min, fixed, permeabilized and stained for Venus (GFP) or an early endosome marker (EEA1) and then imaged on a confocal microscope. We quantified receptor endocytosis by measuring the ratio of Venus and EEA1 positive to total EEA1 positive endosomes. Dose-response curves following 5 min of agonist treatment showed an expected lower potency for PD149163 when compared to neurotensin (EC50, 4.03 nM and 465 nM for neurotensin and PD149163, respectively, Figure 2F), demonstrating that monitoring endogenous NTSR1-Venus receptor endocytosis could differentiate NTSR1 agonists. Since the two agonists elicited a similar response at a 1 μM concentration, we used this concentration to evaluate the kinetics of receptor endocytosis. Time-dependent changes in receptor distribution demonstrated that NTSR1-Venus redistribution to endosomes was rapid, occurring within the first 5 min (98.5% ± 1.29 or 92.7% ± 6.9 for neurotensin and PD149163, respectively) of agonist treatment (Figure 2G). Whereas neurotensin-induced endocytosis began decreasing after 10 min, the amount of NTSR1-Venus detected in early endosomes from neurons treated with PD149163 remained stable, leading to a significantly higher percentage of endosomes containing NTSR1 after 60 min (Figures 2G,H). Together, these results demonstrate distinct potencies and kinetics of the two agonists on NTSR1-Venus receptor internalization which is detectable in living neurons.

Having established evidence supporting functional NTSR1-Venus receptor signaling (Gq/Ca2+) and receptor endocytosis we next evaluated behavioral responses in these animals. Previous reports have demonstrated that systemic administration of NTSR1 agonists induces hypolocomotion (Vadnie et al., 2014). First, when animals were not yet habituated to the activity chambers and only received saline injections, we observed somewhat higher activity counts for *Ntsr1^Venus/Venus^* compared to *Ntsr1^WT/WT^* animals (Supplementary Figure 1). Upon intraperitoneal administration of the brain penetrable *Ntsr1* agonist, PD149163, for both genotypes we saw a dose-dependent decrease in total distance traveled over 2 h as compared to saline animals. A significant difference at 0.2 mg/kg and a trend that was not significant at higher doses (0.5, 1, or 5 mg/kg) was observed between *Ntsr1^Venus/Venus^* and *Ntsr1^WT/WT^* animals (Figure 2I). Notably, saline responses in mice already habituated to the activity chambers were similar in *Ntsr1^Venus/Venus^* and *Ntsr1^WT/WT^* animals. We next used 0.5 mg/kg since it was the lowest concentration of PD149163 capable of inducing the hypolocomotor effect without significant differences between genotypes. In this cohort, 0.5 mg/kg PD149163 induced NTSR1 mediated hypolocomotion to a similar extent in *Ntsr1^Venus/Venus^* and *Ntsr1^WT/WT^* animals albeit with a statistically insignificant slower onset (Figure 2J) but comparable total distance traveled (Figure 2K). These data suggest that NTSR1-Venus signaling, and function appear essentially intact, with however slightly lower responses to agonist treatment.

### NTSR1-Venus tissue distribution in the mouse brain

**FIGURE 3:**
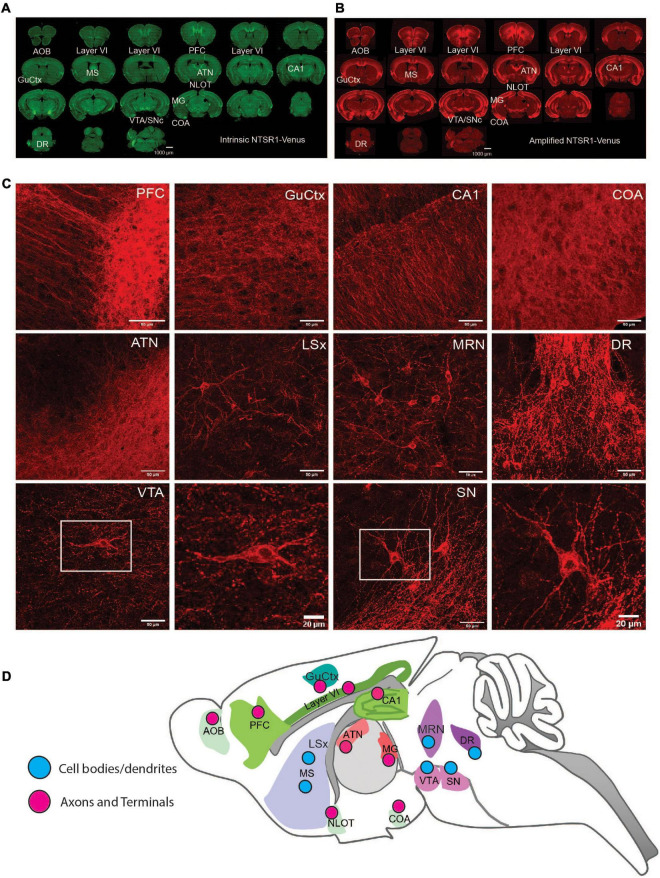
NTSR1-Venus expression in the mouse brain. **(A,B)** Epifluorescence microscope images at macro-level. Annotated overview of the highest NTSR1-Venus expressing brain regions (see Table 2). Scale bars are 1000 μm. **(A)** Green, intrinsic Venus. **(B)** Red, NTSR1-Venus is amplified with Anti-Venus antibody. Complete expression pattern is indicated in Table 2 and visualized in the 3D reconstruction of the amplified NTSR1-Venus **(B)** brain sections in Supplementary Video 3 (*n* = 3 *Ntsr1^Venus/Venus^* animals). **(C)** Confocal imaging of regions with distinct NTSR1-Venus subcellular expression. Scale bars are either 50 μm for macro views or 20 μm for magnified views of SN and VTA. **(D)** Scheme depicts map of subcellular expression pattern (cell bodies/dendrites or axons and terminals) for NTSR1-Venus in regions which highly express NTSR1-Venus. Brain region annotations: AOB, accessory olfactory bulb; ATN, anterior group of the dorsal thalamus; Amd, Anteromedial nucleus-dorsal; COA, Cortical amygdalar area; CA1, CA1 hippocampal region; CA2, CA2 hippocampal region; DG, dentate gyrus; DR, dorsal raphe nucleus; GuCtx, gustatory cortex; IFL, infralimbic area; LSx, lateral septal complex; MS, medial septum; MG, medial geniculate; MRN, midbrain reticular nucleus; NLOT, nucleus of the lateral olfactory tract; PFC, prefrontal cortex; SN, substantia nigra; VTA, ventral tegmental nucleus.

Once NTSR1-Venus animals were found to have functional receptors, we next examined where NTSR1-Venus is observable in tissues. To this end, we fixed, permeabilized and stained adult tissue sections for Venus (Anti-Venus) in a separate channel from Venus to be able to compare the intrinsic (non-amplified) (Figure 3A) and amplified NTSR1-Venus (Figure 3B) signals between *Ntsr1^Venus/Venus^*, *Ntsr1^Venus/WT^* to the background level in *Ntsr1^WT/WT^* animals (Supplementary Figure 2). At regional level, NTSR1 expression in olfactory, cortical, septal, hippocampal, thalamic, and midbrain nuclei were already observable using the Venus-intrinsic fluorescent signal and was further enhanced with antibody-amplified signal. We collected samples from 16 peripheral tissues including liver, heart, intestine, stomach, and colon but NTSR1-Venus was not reliably detected in any of the collected sections (*data not shown*). We also collected various central nervous system tissues including dorsal root ganglion and trigeminal ganglion. Intrinsic and amplified NTSR1-Venus was weakly detected in trigeminal ganglion and only amplified signal was detected in dorsal root ganglion (*data not shown*).

**TABLE 2:** Expression of NTSR1-Venus in adult mouse brain.

Abbreviations	Region	Subregion	Intensity
**OLF**	**Olfactory areas**		

MOB		Main olfactory bulb	2
AOB		Accessory olfactory bulb	3

**CTX**	**Cortex**		

ACA		Anterior cingulate area	
		I–V	0.5
		VI	1
PL		Prelimbic area	
		I–V	1
		VI	3
SS		Somatosensory areas	
		I–V	1
		VI	1
GU		Gustatory areas	
		I–V	2
		VI	1
VISC		Visceral area	
		I–V	2
		VI	3
MO		Somatomotor areas	
		I–V	1
		VI	1
AUD		Auditory areas	
		I–V	1
		VI	3
VIS		Visual areas	
		I–V	1
		VI	1
ILA		Infralimbic area	
		I–V	2
		VI	3
ORB		Orbital area	
		I–V	1
		VI	2
AI		Agranular insular area	
		I–V	1
		VI	3
RSP		Retrospinal area	
		I–V	1
		VI	0.5
PTLp		Posterior parietal association area	
		I–V	1
		VI	3
Tea		Temporal association areas	
		I–V	2
		VI	3
PERI		Perihinal area	
		I–V	1
		VI	3
ECT		Ectorhinal area	
		I–V	1
		VI	3
ENT		Entorhinal areas	
		I–V	1
		VI	3
**HPF**	**Hippocampus**		
CA1		CA1	2
CA2		CA2	0
CA3		CA3	1
DG		Dentate gyrus (po)	2
SUB		Subiculum	1.5
PAR		Parasubiculum	0
POST		Postsubiculum	0.5
**CTXsp**	**Cortical subplate**		
CLA		Claustrum	0
EP		Endopiriform nucleus	2
BLA		Basolateral amygdalar nucleus	0
BMA		Basomedial amygdalar nucleus	1
PA		Posterior amygdalar nucleus	0
**STR**	**Striatum**		
STRd		Striatum dorsal region	0.5
STRv		Striatum ventral region	1
ACB		Nucleus accumbens	0.5
OT		Olfactory tubercle	0.5
LSX		Lateral septal complex	1
CEA		Central amygdalar nucleus	2
IA		Intercalated amygdalar nucleus	0.5
AAA		Anterior amygdalar area	1
MEA		Medial amygdalar nucleus	0
**PAL**	**Pallidum**		
PALd		Dorsal	1
PALv		Ventral	2
PALm		Medial (septum) or MS	2
PALc		Caudal	0.5
BST		Bed nuclei stria terminalis	1
**BS**	**Brain stem**		
**TH**	**Thalamus**		
SMT		Submedial nucleus	0
Amd		Anteromedial nucleus, dorsal part	2.5
ATN		Anterior group of the dorsal thalamus	3
VPM		Ventral posteromedial nucleus	0
RT		Reticular nucleus	0
MTN		Midline group of dorsal thalamus	0
HB		Habenula	1
MG		Medial geniculate	3
LAT		Lateral group of dorsal thalamus	2
**HY**	**Hypothalamus**		
PVZ		Periventricular zone	1
PVR		Periventricular region	1.5
MEZ		Hypothalamic medial zone	1.5
LZ		Hypothalamic lateral zone (ZI)	2
MPN		Median preoptic nucleus	1
**MB**	**Midbrain-motor**		
SNc		Substantia Nigra c	3
SNr		Substantia Nigra r	1
VTA		Ventral tegmental area	3
EW		Edinger-Westphal nucleus	2
RR		Midbrain reticular nucleus retrorubral area	2
PAG		Periaqueductal gray	2
**MBsta**	**Midbrain, behavioral state**		
RAmb		Midbrain raphe nuclei	0.5
DR		Dorsal Raphe nucleus	2
RN		Red nucleus	0.5
MRN		Midbrain reticular nucleus	1
**HB**	**Hindbrain**		
P	PONS	CS, PB, SOC	1
MY	Medulla	MARN, SUV, RM	2
**OLF**	**Olfactory areas**		
PAA		Piriform-amygdalar area	1
COA		Cortical amygdalar area	3
PiR		Piriform area	0.5
NLOT		Nucleus of the lateral olfactory tract	3

Amplified NTSR1-Venus intensity was assessed in *n = 3 Ntsr1^Venus/Venus^* animals. Brain areas were annotated according to Allen brain atlas classification. NTSR1-Venus expression was semi-quantified by comparing fluorescence intensity across areas to other brain regions within the same section. No expression (0), low expression (1), moderate expression (2), high expression (3).

At regional level, slide scanner imaged brain sections were utilized to fully reconstruct the whole brain in 3D revealing the most prominent expressing regions in cortex, thalamus and midbrain (Supplementary Video 3). For a detailed analysis of NTSR1-Venus at regional brain level, amplified fluorescent signal was semi-quantified across 92 regions (Table 2). The regions with moderate to high expression include the cortical layer 6 where NTSR1 is visible throughout the brain, and all cortical layers in the infralimbic area (IFL) of the prefrontal cortex (PFC), the gustatory cortex (GuCtx), and hippocampal areas including CA1. NTSR1 expression was also high in thalamic nuclei including anterior groups of the dorsal thalamus (ATN) and medial geniculate (MG). Midbrain areas comprising dorsal raphe nucleus (DR), substantia nigra (SN), and ventral tegmental area (VTA) also contained high NTSR1 expression. Finally, striatal related areas consisting of medial septum (MS) and olfactory areas like the nucleus of the lateral olfactory tract (NLOT), accessory olfactory bulb (AOB), and cortical amygdalar area (COA) also contained higher levels of NTSR1-Venus fluorescence than other brain regions examined.

To examine subcellular expression, we imaged amplified sections on a confocal microscope (Figure 3C). We observed two types of NTSR1-Venus expression patterns which we categorized as cell bodies/dendrites (neuronal soma and processes) or axons/terminals (fiber/projection). The former pattern, in which neuronal soma were evident, was observed in CA1, CA3, dentate gyrus, MS, lateral septal complex, DR, SN, and VTA. The latter pattern, a more diffuse unstructured signal, was observed in PFC, GuCtx, COA, and anterior group of the dorsal thalamus. A schematic summarizes the above-described brain mapping of NTSR1-Venus regional and subcellular distribution (Figure 3D). These results suggest that adult NTSR1-Venus animals possess expected tissue distribution (cortical, thalamic, midbrain, etc.) as predicted by the literature and allows for subcellular localization of the receptor in brain sections.

### NTSR1-Venus localizes to dopaminergic neurons

**FIGURE 4:**
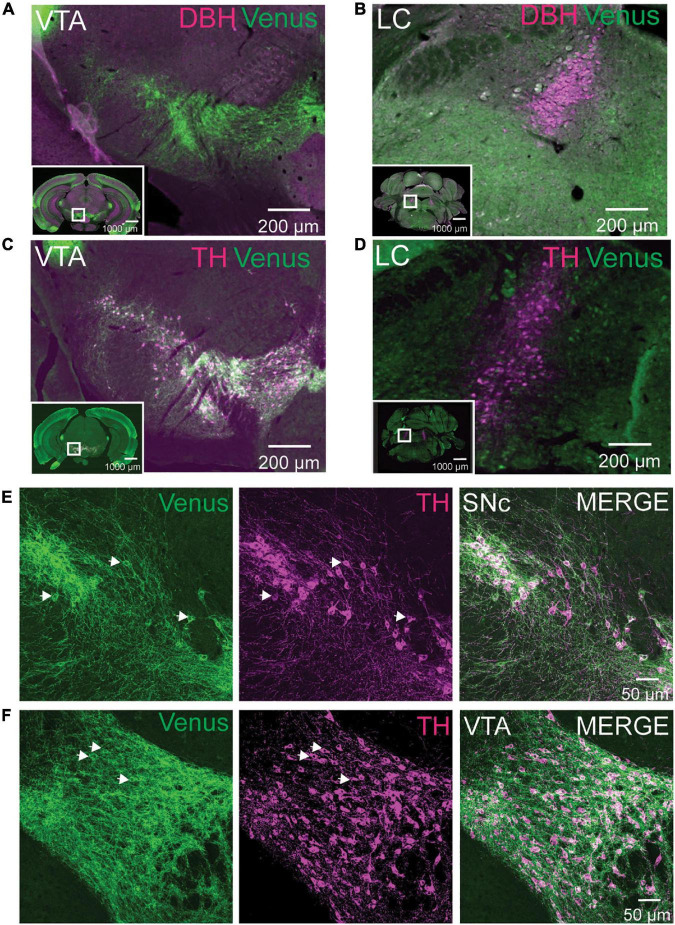
NTSR1-Venus neurons are dopaminergic VTA neurons. **(A,B)** Tissue sections from *Ntsr1^Venus/Venus^* animals were stained with anti-Venus (green) or noradrenergic cell marker anti-dopamine-β-hydroxylase (DBH) shown in magenta. Neurons in midbrain dopaminergic regions ventral tegmental area (VTA) **(A)**, and noradrenergic locus coeruleus (LC) **(B)** are shown. **(C,D)** Tissue sections from *Ntsr1^Venus/Venus^* animals were stained with anti-Venus (green) or dopaminergic cell marker anti-tyrosine hydroxylase (TH) shown in magenta. Co-labeling was assessed in VTA **(C)** and LC **(D)**. For panels **(A–D)**, co-labeled cells are shown in white. Scale bars are 1000 μm for the macro view (insets) and 200 μm for the magnified view. White boxes found on insets highlight areas of magnification. Images were acquired with epifluorescence on an Olympus VS120 slide scanner. **(E,F)** Tissue sections from *Ntsr1^Venus/Venus^* animals were stained with anti-Venus (green) or dopaminergic cell marker anti-TH (magenta). Confocal images of co-labeled NTSR1-Venus and dopaminergic neurons in substantia nigra compacta (SN) **(E)** and VTA **(F)** are shown. Scale bars are 50 μm. **(A–F)** Images are representative of *n* = 3 *Ntsr1^Venus/Venus^* animals.

Our brain mapping identified enrichment of NTSR1-Venus positive neurons in the midbrain. Based on previous *in situ* hybridization reports (Nicot et al., 1995), we reasoned that neurons in the SN and VTA were likely dopaminergic and not noradrenergic which heavily concentrates in the locus coeruleus (LC). To test this hypothesis, we first immunostained adult *Ntsr1^Venus/Venus^* brain sections for Venus and dopamine beta-hydroxylase (DBH) to identify noradrenergic cells using epifluorescence microscopy. We did not observe any colocalization between DBH and Venus in the VTA, where cells were NTSR1-Venus positive and DBH negative (Figure 4A; Supplementary Video 1) or in the LC where DBH positive neurons were Venus negative (Figure 4B). However, when we stained brain sections for Venus and tyrosine hydroxylase (TH), a dopaminergic neuron marker, TH colocalized with Venus in the VTA (Figure 4C) but not in LC (Figure 4D). When slide scanner imaged brain sections were used to 3D reconstruct the whole brain, we noticed that the VTA and SN areas were the most enriched areas for Venus/TH neurons (Supplementary Video 4). Upon closer examination of the dual Venus/TH-stained brain sections using confocal microscopy, we observed colocalization on soma and processes in SN (Figure 4E) and VTA (Figure 4F) neurons. These results suggest that NTSR1-Venus mice show enriched expression in dopaminergic neurons within the midbrain.

### Real-time imaging of NTSR1 localization and trafficking at subcellular resolution in intact brain slices

**FIGURE 5:**
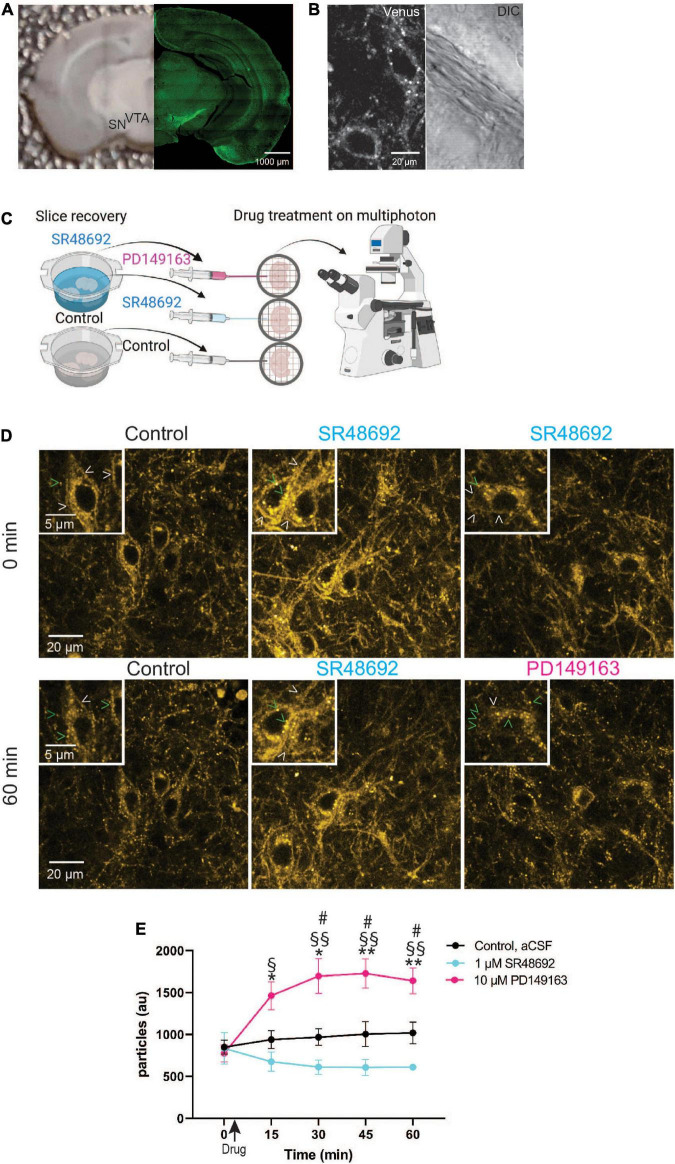
Monitoring GPCR activation in dopaminergic midbrain neurons in acute slices using NTSR1-Venus animals. **(A)** Shown is a representative image of an acute slice prepared from *Ntsr1^Venus/Venus^* animals used for multiphoton imaging (left) and an image of NTSR1-Venus intrinsic expression (right). Scale bars are 1000 μm. **(B)** A representative high magnification two-photon image shows healthy living NTSR1-Venus cell bodies in acute slices prepared from control *Ntsr1^Venus/Venus^* animals. Venus expression is shown (left) and DIC is shown (right). Scale bars are 20 μm. **(C)** Experimental schematic shows during slice recovery, 1/3 of slices were incubated in control cutting solution, 2/3 of slices were incubated in 1 μM SR48692, and following 0 min image acquisition, either aCSF (black), 1 μM SR48692 (blue), or 10 μM PD149163 (pink) was added to the aCSF flowing in the perfusion system. Scheme created with www.biorender.com. **(D)** Representative multiphoton images of acute slices containing the VTA from *Ntsr1^Venus/Venus^* animals used to measure NTSR1-Venus internalization. Slices were first incubated with either cutting solution or 1 μM SR48692. Baseline images were obtained and following image acquisition the indicated drug was added—control (aCSF), 1 μM SR48692 or 10 μM PD149163. Scale bars are 20 μm and 2 μm (insets). Arrowheads indicate representative puncta (green) or membrane (white) localized receptor. **(E)** Internalization of NTSR1-Venus was quantified as average number of particles per image. Images are representative of *n* = 4–5 acute slices per condition from *Ntsr1^Venus/Venus^* animals. Graph depicts the mean ± SEM. *Indicates significance compared to PD149163 at 0 min, # comparison between PD149163 and control, §comparison between PD149163 and SR48692. *^#,§^ *p* < 0.05, **^§§^ *p* < 0.01 *via* 2way ANOVA [significant time effect *F*_(2_._406_,_24_._06)_ = 7.457; *p* = 0.0019], significant drug effect *F*_(2_,_10)_ = 11.13; *p* = 0.0029 with a significant interaction *F*_(8_,_40)_ = 11.41; *p* < 0.0001.

Studying GPCR trafficking in living tissues has been a highly desired yet unfulfilled goal. The robust labeling of NTSR1-Venus on soma of dopaminergic neurons motivated us to ask whether we could use these animals to monitor receptor endocytosis in living tissue sections? We prepared acute coronal slices containing SN and VTA (Figure 5A) from 5-week-old *Ntsr1^Venus/Venus^* animals for multiphoton imaging. In untreated slices, we observed that basal NTSR1-Venus was visible at the membrane and punctate internal structures throughout soma and processes (Figure 5B) suggesting that endogenous neuropeptide/neurotransmitter release could have promoted receptor endocytosis.

To test whether NTSR1 receptors are indeed being bound by endogenous ligand in acute slice, we compared NTSR1 distribution in slices treated with the NTSR1 specific antagonist, SR48692 (Gully et al., 1993). In these experiments, slices were prepared as before and a third of them were placed in cutting solution (control) whereas the rest of the slices were placed in cutting solution (see Section “Materials and methods”) containing 1 μM SR48692 for the duration of slice recovery. Following slice recovery, VTA/SN-containing slices were placed in the imaging chamber with circulating aCSF and a baseline image (0 min) was acquired. Immediately following image acquisition, either drug was added to the circulating aCSF or for control slices, nothing was added. For slices which had recovered in 1 μM SR48692, either 1 μM SR48692, or 10 μM PD149163 was added to the slice (Figure 5C). We began each 60-min imaging session with a baseline Z-stack image taken at 0 min and subsequent Z-stack acquired images were taken every 15 min. In control slices, NTSR1-Venus expression was observed at plasma membrane and internal punctate structures for the duration of imaging (Figure 5D). In antagonist recovered and treated slices, NTSR1-Venus remained visible at internal punctate structures as well as plasma membrane. Remarkably, addition of 10 μM PD149163 onto slices preincubated with antagonist during slice recovery, enabled the observation of agonist-induced NTSR1-Venus puncta throughout soma and processes. NTSR1-Venus puncta were then quantified in the acquired image projections by counting vesicle-sized particles (Figure 5E). In control slices, a similar number of puncta were visible at the start (0 min) and at the end (60 min) of imaging. Antagonist-only treated slices showed a trend of decreasing puncta across imaging time perhaps suggesting increased membrane localized receptors. In 10 μM PD149163 stimulated slices, vesicle-sized particle counts were significantly higher than controls at 30, 45, and 60 min and significantly higher than antagonist-only treated slices at 15, 30, 45, and 60 min. Taken together, these results indicate that our endogenous labeling strategy enables the subcellular localization and trafficking of NTSR1 to be visualized directly, and with high spatiotemporal resolution, in intact brain slices.

## Discussion

G protein-coupled receptors are low abundance membrane-bound proteins that have major regulatory physiological functions. However, their low detectability has made it difficult to fully characterize their role(s) in physiologically relevant models. Development of GPCR therapeutics would benefit from the study of GPCRs in their native environment. Yet, high affinity specific antibodies to detect endogenous receptors are lacking for many members of the family. Knock-in animal approaches such as Rhodopsin-GFP (Chan et al., 2004), DOR-eGFP (Scherrer et al., 2006), MOR-mCherry (Erbs et al., 2015), NOP-eGFP (Ozawa et al., 2015), KOR-tdTomato or KtdT (Chen et al., 2020), MOR-Venus (Ehrlich et al., 2019), GPR88-Venus (Ehrlich et al., 2018), ACKR3-Venus (Ehrlich et al., 2021), have demonstrated that a fluorescent protein can be added to the C-terminus of GPCRs yielding a more readily detectable receptor, expressed under endogenous transcriptional control, that retains expected tissue distribution and function. Here, we report the characterization of new fluorescent receptor knock-in mice, the NTSR1-Venus.

Modification of a GPCR by addition of an epitope or a fluorescent protein sequence can potentially change the receptor’s activity. Here, we demonstrate that the normal activation and internalization of NTSR1-Venus is maintained. NTSR1-Venus had similar potencies to WT receptors for neurotensin and PD149163 in Gq activation in HEK293 cells and in intracellular Ca^2+^ signaling assays in hippocampal neurons. We also show in neurons that basal NTSR1-Venus traffics to plasma membrane allowing for agonist mediated redistribution to endosomes. Moreover, in behavioral assays, we found that activation with PD149163 reduces locomotor behavior similarly to what is observed in WT animals.

Additionally in locomotion assays, we show NTSR1-Venus animals are less sensitive to PD149163 than WT animals indicating that future behavioral assays will require careful consideration about the appropriate concentration to use. This may be related to the lower efficacy of NTSR1-Venus to activate Gq observed in the cell-based assay. Alternatively, the decrease in agonist-induced behavioral response may arise from distinct receptor degradation or recycling in NTSR1-Venus animals. It is intriguing that owing to a difference in their C-termini, in contrast to NTSR2, NTSR1 receptors have been reported not to recycle (Botto et al., 1998; Martin et al., 2002) and to undergo lysosomal degradation instead (Vandenbulcke et al., 2000). Going forward, it will be interesting to utilize recombinant systems and NTSR1-Venus animals to investigate further the life cycle of NTSR1 receptors.

In the present study, we mapped the receptor throughout the central nervous system. Intrinsic fluorescence of NTSR1-Venus was sufficient to allow for direct visualization of the Venus-fused receptors on neurons and antibody-mediated amplification of the Venus signal allowed a greater cellular resolution. In general, we observed protein in the reported RNA transcript expression pattern (Nicot et al., 1994; Alexander and Leeman, 1998; Lein et al., 2007) with strong expression in midbrain (VTA and SN), olfactory areas, thalamic nuclei, and moderate expression in select pallidum, amygdala, cortical, hippocampal, hypothalamic, and midbrain areas.

For a few brain regions, the observed protein expression differed from other reports. Transcripts have been detected in the infralimbic cortex of adult rodents by single-cell RNA-seq analysis (Tasic et al., 2016) and some ISH reports (Nicot et al., 1994; Alexander and Leeman, 1998) but not others (Lein et al., 2007). This has led to some discussion about whether the observed expression of *Ntsr1* in cortical layer 6b pyramidal cells of *Ntsr1-Cre* animals (Sundberg et al., 2018) is from active *Ntsr1* transcripts or a transgene recombination event leftover from development (Schroeder and Leinninger, 2018). Our findings support *Ntsr1* transcript and protein as being expressed on fibers and terminals in the prefrontal cortex and cortical layer 6. Also, several studies have indicated expression of *Ntsr1* transcripts in habenula (Moyse et al., 1987; Nicot et al., 1994; Alexander and Leeman, 1998) and a single immunodetection study described NTSR1 in medial but not lateral habenula (Boudin et al., 1996). In habenula, we found negligible *Ntsr1* transcript levels, which may be expected from microdissected tissue-based RT-PCR assays, and NTSR1-Venus protein expression from brain section imaging was low but slightly more evident in lateral than medial habenula. Finally, the central amygdala has been reported to contain *Ntsr1* transcripts (Alexander and Leeman, 1998) but not protein (Boudin et al., 1996). We observed moderate expression of NTSR1-Venus suggesting that the protein is also present at this brain site. Future experiments could examine whether transcript and protein reside in the same neurons by conducting transcript analysis in brain sections from NTSR1-Venus animals.

NTSR1-Venus receptor somato-dendritic localization was most highly detectable in midbrain regions including VTA, SN, periaqueductal gray, dorsal raphe, midbrain reticular nucleus, and magnocellular reticular nucleus. These areas are components of the reticular formation, a broadly defined group of nuclei that have roles in behavioral arousal and consciousness through connectivity between the brain stem and telencephalon. Our observation that NTSR1-Venus colocalizes with tyrosine hydroxylase labeled dopaminergic neurons agrees with previous reports (Nicot et al., 1995; Yamada et al., 1995). In fixed sections and live VTA slice preparations, we observed NTSR1-Venus along neuronal membranes, with a punctate pattern previously termed as “hot spots” (Boudin et al., 1996) where receptors congregate along somato-dendritic sites. These collections of receptors are theorized to be receptors that have recycled back to the membrane or a localized storage of NTSR1 receptors that could be mobilized to respond upon NT stimulation (Boudin et al., 1996). Future studies may utilize NTSR1-Venus mice to characterize these receptor pools.

In acute VTA slice preparations, we also observed untreated NTSR1-Venus at the plasma membrane and in puncta following slice recovery. NTSR1-Venus was stabilized at the plasma membrane in slices treated with antagonist. Interestingly, delta opioid receptor (DOR) electrophysiological studies using DOR-eGFP animals, previously reported that a DOR-eGFP subcellular localization is altered during slice recovery and required decreased electrical activity by substitution of sodium chloride with potassium gluconate to restore plasma membrane expression, as the altered intracellular localization was not reversible by its antagonist naltrexone (Rezai et al., 2013). Although here, we cannot rule out that other chemical factors induced by cutting may have contributed to punctate NTSR1 localization, the agonist mediated redistribution was improved following antagonist treatment suggesting that the subcellular distribution was likely due to local neuropeptide release.

The possibility of observing GPCR activities in living tissues has been an open question in the field for some time. In this study, we were able to observe NTSR1-Venus receptor trafficking in real-time. Direct observation of NTSR1-Venus will enable future studies to examine NTSR1’s neuromodulation of dopamine and signaling properties at receptor level and may contribute to drug discovery efforts in Parkinson’s disease, schizophrenia and addiction. For example, following compound screening, top hit compounds could be evaluated in cultured neurons or acute slices from NTSR1-Venus animals to test their ability to engage the receptor and promote endocytosis. More broadly, because NTSR1 is pleiotropic (Besserer-Offroy et al., 2017) and robustly internalizes upon agonist stimulation, studies using NTSR1-Venus may improve our understanding of how GPCRs respond to distinct pharmacological stimuli and how receptor trafficking and endocytosis itineraries influence signaling outcomes. Together, this work establishes the NTSR1-Venus mouse as an excellent model for the study of GPCR trafficking, internalization, and signaling in living neurons.

## Data availability statement

The original contributions presented in this study are included in the article/Supplementary material, further inquiries can be directed to the corresponding authors.

## Ethics statement

This animal study was reviewed and approved by Canadian Council of Animal Care and Institutional Animal Care and Use Committee approved protocol (AN185688).

## Author contributions

AE, PC, SS, and KB designed the experiments. AE, PC, SS, SW, DD, AM, and KB performed the experiments. AE, PC, SW, DD, and AM analyzed the data. AE, PC, SS, MZ, KB, MB, and BK drafted and edited the manuscript. AE, MZ, MB, and BK funded the work. AE, MB, BK, MZ, and KB conceptualized the work. All authors contributed to the article and approved the submitted version.

## Footnotes

1
http://www.phenomin.fr/en-us/

2
http://atlas.brain-map.org/

## Supplementary material

Supplementary Figure 1 Spontaneous locomotor activity. Saline injected animals were not habituated to activity boxes and allowed to move freely for 150 min. Data shown in 10-min bins. **p* < 0.05 *via* 2way RM ANOVA with a Bonferroni’s *post-hoc* test [significant effect by time *F*_(14,238)_ = 50.54; *p* < 0.0001], significant effect by genotype *F*_(1,17)_ = 8.534; *p* = 0.0095 with a significant interaction *F*_(14,238)_ = 1.909; *p* = 0.0262.

Supplementary Figure 2 Intrinsic and amplified NTSR1-Venus expression in mouse midbrain sections. **(A–C)** NTSR1-Venus expression is visible according to gene dosage. **(A)** *Ntsr1*^WT/WT^**. **(B)** *Ntsr1*^Venus/WT^**. **(C)** *Ntsr1^Venus/Venus^*.

Supplementary Video 1 Live confocal imaging captures NTSR1-Venus internalization in hippocampal neurons treated with 1 μM PD149163. NTSR1-Venus trafficking was monitored for 20 min following agonist addition. 60x objective on a Zeiss LSM710 laser scanning confocal microscope.

Supplementary Video 2 Live confocal imaging of NTSR1-Venus hippocampal neurons treated with vehicle. NTSR1-Venus trafficking was monitored for 20 min following vehicle addition. 60x objective on a Zeiss LSM710 laser scanning confocal microscope.

Supplementary Video 3 Amplified NTSR1-Venus expression in the 3D reconstructed mouse brain. Whole brain 3D reconstruction of brain sections immunostained for Venus (red).

Supplementary Video 4 Amplified NTSR1-Venus expression colocalizes with dopaminergic cells. 3D reconstructed mouse brain from brain sections immunostained for Venus (green) and dopaminergic cells detected with anti-tyrosine hydroxylase (red).
