# Supplementary figures and images for: Visualization of real-time receptor endocytosis in dopamine neurons enabled by NTSR1-Venus knock-in mice
Source: Front Cell Neurosci. 2022 Nov 29;16:1076599. doi: 10.3389/fncel.2022.1076599 (PMC9745132; doi:10.3389/fncel.2022.1076599)

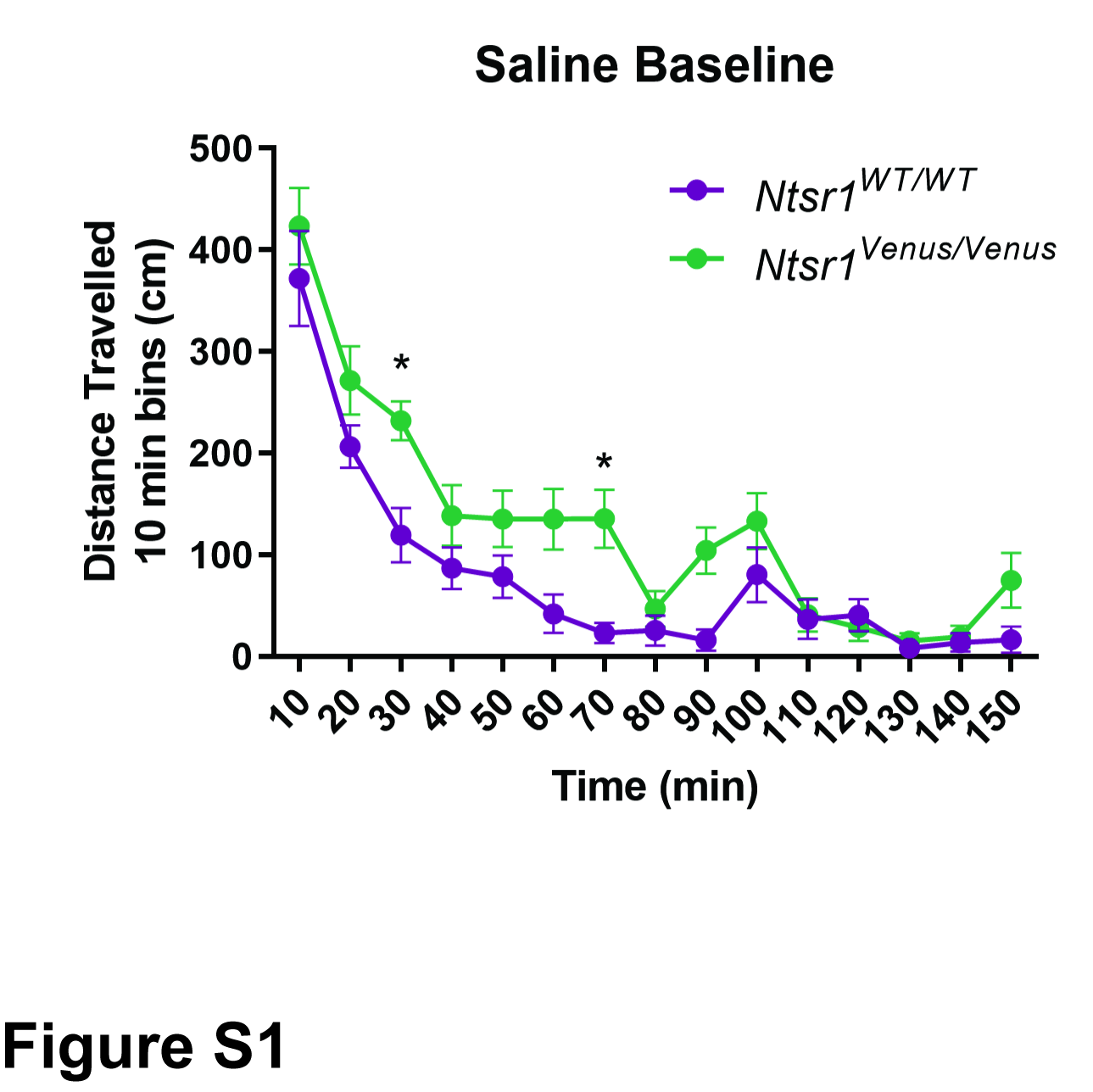

Supplement: Supplementary Figure 1 — Spontaneous locomotor activity. Saline injected animals were not habituated to activity boxes and allowed to move freely for 150 min. Data shown in 10-min bins. *p < 0.05 via 2way RM ANOVA with a Bonferroni’s post-hoc test [significant effect by time F(14,238) = 50.54; p < 0.0001], significant effect by genotype F(1,17) = 8.534; p = 0.0095 with a significant interaction F(14,238) = 1.909; p = 0.0262. [file Image_1.TIF]

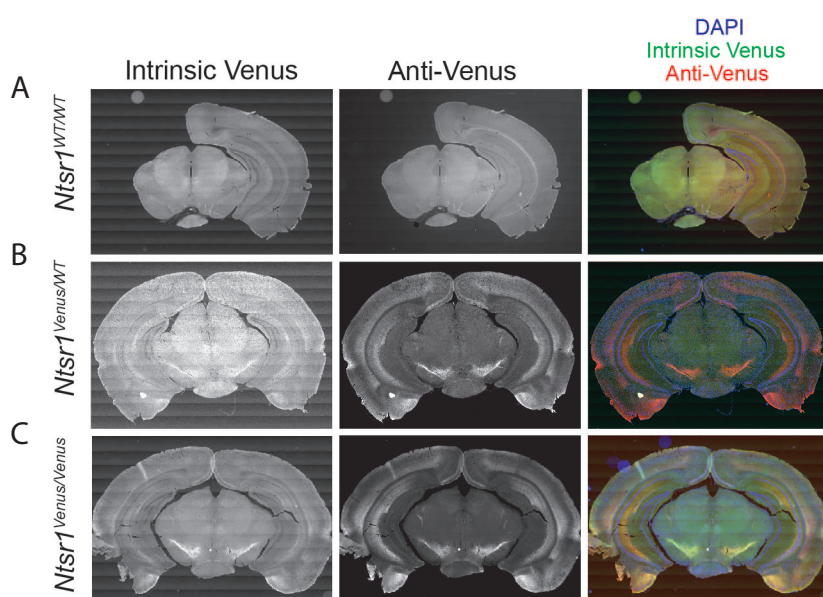

Figure S2

Supplement: Supplementary Figure 2 — Intrinsic and amplified NTSR1-Venus expression in mouse midbrain sections. (A–C) NTSR1-Venus expression is visible according to gene dosage. (A) Ntsr1WT/WT. (B) Ntsr1Venus/WT. (C) Ntsr1Venus/Venus. [file Image_2.pdf]
